# Multi-Server Multi-User Multi-Task Computation Offloading for Mobile Edge Computing Networks

**DOI:** 10.3390/s19061446

**Published:** 2019-03-24

**Authors:** Liang Huang, Xu Feng, Luxin Zhang, Liping Qian, Yuan Wu

**Affiliations:** College of Information Engineering, Zhejiang University of Technology, Hangzhou 310023, China; xfeng_zjut@163.com (X.F.); lxzhang_zjut@163.com (L.Z.); lpqian@zjut.edu.cn (L.Q.); iewuy@zjut.edu.cn (Y.W.)

**Keywords:** mobile edge computing, computation offloading, deep reinforcement learning

## Abstract

This paper studies mobile edge computing (MEC) networks where multiple wireless devices (WDs) offload their computation tasks to multiple edge servers and one cloud server. Considering different real-time computation tasks at different WDs, every task is decided to be processed locally at its WD or to be offloaded to and processed at one of the edge servers or the cloud server. In this paper, we investigate low-complexity computation offloading policies to guarantee quality of service of the MEC network and to minimize WDs’ energy consumption. Specifically, both a linear programing relaxation-based (LR-based) algorithm and a distributed deep learning-based offloading (DDLO) algorithm are independently studied for MEC networks. We further propose a heterogeneous DDLO to achieve better convergence performance than DDLO. Extensive numerical results show that the DDLO algorithms guarantee better performance than the LR-based algorithm. Furthermore, the DDLO algorithm generates an offloading decision in less than 1 millisecond, which is several orders faster than the LR-based algorithm.

## 1. Introduction

The last decade has witnessed how mobile devices and mobile applications have become an indispensable part of peoples’ lives. Mobile devices provide a wide range of digital services, such as map navigation, language recognition, web browsing, and so on. Besides being a means of phone calls and content consumption, mobile devices tend to be platforms that assist people to accomplish more online tasks as a complement to desktop computers and laptops. These tasks require a large amount of computing resources and stringent quality of service (QoS), e.g., Augmented Reality (AR) applications [1], Vehicular ad-hoc networks (VANETs) [2], and cloud gaming [3]. Due to limited computation resources and the size-constrained batteries of mobile devices, computationally intensive tasks are offloaded to remote computational servers, which then transfer computing results back to the mobile devices, known as cloud computing [4]. However, this approach suffers high latency and unstable QoS due to data propagation and routing between mobile devices and remote cloud servers. Although different wireless communication technologies [5,6,7] and data transmission scheduling schemes [8,9,10,11] have been developed in the past decades, the QoS is slightly improved due to the long-distance transmissions between mobile devices and remote cloud servers. Recently, mobile edge computing (MEC) network is proposed to deploy multiple edge servers close to mobile devices. Mobile devices in MEC networks can efficiently offload their tasks to nearby edge servers and receive immediate feedback after processing, so as to improve the QoS. For example, after the emergence of Internet of Things (IoT), more and more sensors are connected to MEC networks. The massive measured data can be offloaded to edge servers with low processing latency, which can also extend the computation power of IoT sensors [12]. In the coming fifth-generation (5G) mobile network, the deployment of ultra-dense small cell networks (UDNs) is envisaged [13]. There are going to be multiple edge servers within the wireless communication range of each mobile device, so as to provide sufficient edge servers and communication capacity for MEC networks. However, it is challenging to make computation offloading decisions when multiple edge servers and mobile devices are available in MEC networks. For example, *whether a computing task should be offloaded to edge servers? Which edge server should it be offloaded to?* Different offloading decisions result in different QoS of the MEC networks. Thus, it is important to carefully design computation offloading mechanism for MEC networks.

In MEC networks, computation offloading is challenged by limited computing resources and real-time delay constraint. Different from large-scale cloud computing centers, edge servers are small-scale with limited processing capacity. When lots of tasks being offloaded to the same edge server it causes congestion, resulting in longer processing time delay for all tasks. Therefore, simply offloading a task to its closest edge server may not be a good choice. An offloading decision depends on available computing capacities at local mobile device, edge servers, and cloud servers, along with communication capacity. Computation offloading in MEC networks is widely studied by using convex optimization [14] and linear relaxation approximation [15,16], which takes too long time to be employed in MEC networks with dynamic computation tasks and time-varying wireless channels. An efficient and effective computation offloading policy for multi-server multi-use MEC networks is still absent.

In this paper, we consider a MEC network with multiple edge servers and one remote cloud server, where multiple wireless devices (WDs) offload their tasks to edge/cloud servers. We investigate both a linear programing relaxation-based (LR-based) algorithm and a heterogeneous distributed deep learning-based offloading (DDLO) algorithm to guarantee QoS of the MEC network and to minimize WDs’ energy consumption. The heterogeneous DDLO algorithm takes advantage of deep reinforcement learning and is insensitive to the number of WDs. It outperforms the LR-based algorithm in terms of both system utility and computing delay.

Deep reinforcement learning has been applied in many aspects, e.g., natural language process [17], gaming [18], and robot control [19]. It uses a deep neural network (DNN) to empirically solve large-scale complex problems. There exist few recent works on deep reinforcement learning-based computation offloading for MEC networks [20,21,22,23]. Huang et al. proposed a distributed computation offloading algorithm based on deep reinforcement learning, DDLO [23], for MEC networks with one edge server and multiple WDs. They take advantage of multiple DNNs with identical network structure and show that the computation delay is independent of the number of DNNs. In this paper, we apply DDLO to MEC networks with multiple servers and multiple WDs and further improve the performance of DDLO by using heterogeneous DNN structures.

### 1.1. Previous Work on Computation Offloading in MEC Networks

Considering a MEC network single edge server, Wei et al. [24] presented an architecture, MVR, to enable the use of virtual resources in edge server to alleviate the resource burden and reduce energy consumption of the WDs. You et al. [25] proposed a framework where a WD can harvest energy from a base station or offload task to it. Muñoz et al. [26] jointly optimized the allocation of radio and computational resource to minimize the WD’s energy consumption. For MEC networks with multiple WDs, Huang et al. [23] proposed a distributed deep learning-based offloading algorithm, which can effectively provide almost optimal offloading decisions for a MEC nework with multiple WDs and single edge server. To get avoid of the curse of dimensionality problem, Huang et al. [27] proposed a deep reinforcement learning-based online offloading (DROO) framework to instantly generate offloading decisions. Chen et al. [28] proposed an efficient distributed computation offloading algorithm which can be used to achieve a Nash equilibrium in multiple WDs scenario.

Considering a MEC network with multiple edge servers, Dinh et al. [16] considered a MEC with multiple edges servers, and proposed two approach, linear relaxation-based approach, and a semidefinite relaxation (SDR)-based approach to minimize both total tasks’ execution latency and WDs’ energy consumption. Authors [29] also considered the case of multiple edge servers and obtain the optimal computation distribution among servers. For multiple-server multiple-user MEC networks, authors [30] proposed a model free reinforcement learning offloading mechanism (Q-learning) to achieve the long-term utilities.

Considering a MEC network with both edge servers and a remote cloud server. Chen et al. [31] studied a general multi-user mobile cloud computing system with a computing access point (CAP), where each mobile user has multiple independent tasks that may be processed locally, at the CAP, or at a remote cloud server. Liu et al. [12] studied an edge server and cloud server to reduce energy consumption and enhance computation capability for resource-constrained IoT devices. Li et al. [32] also studied a computation offloading management policy by jointly processing the heterogeneous computation resources, latency requirements, power consumption at end devices, and channel states. We further categorize all these related works with respect to the number of tasks, WDs, and servers in Table 1.

### 1.2. Our Approach and Contributions in This Paper

In this paper, we consider a network with multiple WDs, multiple edge servers, and one cloud server. Each WD has multiple tasks, which can be offloaded to and processed at edge and cloud servers. To guarantee the QoS of the network and minimize WDs’ energy consumption, we obtain the following results:

1. We model the system utility as the weighted sum of task completion latency and WDs’ energy consumption. To minimize the system utility, we investigate a linear programming relaxation-based (LR-based) algorithm to approximately optimize the offloading decisions for each task of a WD.
2. We extend the DDLO algorithm to multiple-server MEC network. We further propose a heterogeneous DDLO algorithm by generating offloading decisions through multiple DNNs with heterogeneous network structure, which has better convergence performance than DDLO.
3. We provide extensive simulation results to evaluate LR-based algorithm, DDLO algorithm, and heterogeneous DDLO algorithm. Extensive numerical results show that the DDLO algorithms guarantee better performance than the LR-based algorithms.

The rest of the paper is organized as follows. In Section 2, we present the system model and problem formulation. We present an LR-based algorithm in Section 3 and an heterogeneous DDLO algorithm in Section 4. Numerical results are presented in Section 5, and a conclusion is provided in Section 6.

## 2. System Model and Problem Formulation

### 2.1. MEC Network

**Figure 1:**
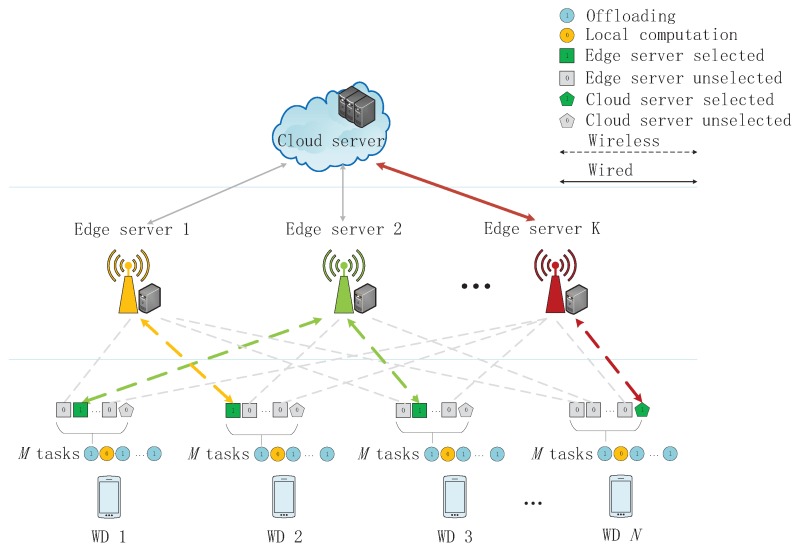
System Model of a multi-server multi-user multi-task mobile edge computing (MEC) network.

In this work, we consider a MEC network composed by one cloud server, *K* edge servers, and *N* wireless devices (WDs), as shown in Figure 1. Without loss of generality, we assume that each WD has *M* independent tasks where each task can be computed by the WD itself or be offloaded to and processed by the edge servers or the cloud server. We denote the set of WDs as N={1,2,…,N}, the set of tasks as M={1,2,…,M}, and the set of servers as K={0,1,2,…,K,K+1}, where server 0 denotes the WD itself and server K+1 denotes the cloud server. Each WD must make decisions on whether remotely processing or locally processing for each of its tasks. We denote $a_{nmk}\in \left\{0,1\right\}$ as the offloading decision that WD *n*’s *m*-th task is assigned to the server *k*, where n∈N, m∈M, and k∈K. Specifically, $a_{nm0}=1$ means that WD *n* decides to locally execute its *m*-th task. Then, we have $a_{nmk}=0,\forall k\in K\setminus \left\{0\right\}$. Overall, every task must be processed by one of those servers (including server 0), as $\sum_{k=0}^{K+1}a_{nmk}=1$, whose exact computing mode depends on

$$\left\{\begin{array}{cc} a_{nm0}=1, & \mathrm{local}\;\mathrm{computing},\\ \sum_{k=1}^{K}a_{nmk}=1, & \mathrm{edge}\;\mathrm{computing},\\ a_{nmk+1}=1, & \mathrm{cloud}\;\mathrm{computing}, \end{array}\right.$$

for any n∈N and m∈M. The detailed operations of communication and computing are illustrated as follows.

### 2.2. Communication Model

Here we study transmission latency and energy consumption due to communications between WDs and servers. We set a tuple $\left(\alpha_{nm},\beta_{nm},\gamma_{nm}\right)$ to represent WD *n*’s *m*-th task, for n∈N, m∈M. Specifically, $\alpha_{nm}$ is the data size, $\beta_{nm}$ is the corresponding size back from the servers, and $\gamma_{nm}$ is the required number of CPU cycles to complete the task. When one of WD *n*’s tasks is offloaded to the edge server k∈K∖{0,k+1}, the uplink and downlink transmission rates between the WD *n* and the edge server *k* are quantified as

(1) $$\begin{array}{c} C_{nk}^{\mathrm{UL}}=B_{nk}^{\mathrm{UL}}\log_{2}\left(1+\frac{P_{n}^{\mathrm{TX}}h_{nk}}{\omega_{0}+\sum_{i\in N\setminus \{n\}}P_{i}^{\mathrm{TX}}h_{is}\sum_{m\in M}a_{imk}}\right), \end{array}$$

(2) $$\begin{array}{c} C_{nk}^{\mathrm{DL}}=B_{nk}^{\mathrm{DL}}\log_{2}\left(1+\frac{P_{k}^{\mathrm{TX}}h_{nk}}{\omega_{0}+\sum_{i\in K\setminus \{k\}}P_{j}^{\mathrm{TX}}h_{nj}\sum_{m\in M}a_{nmj}}\right), \end{array}$$

where $B_{nk}^{\mathrm{UL}}$ and $B_{nk}^{\mathrm{DL}}$ are the uplink and downlink transmission channel bandwidths, $P_{n}^{\mathrm{TX}}$ and $P_{k}^{\mathrm{TX}}$ are the transmission powers of the WD *n* and the edge server *k*, $h_{nk}$ is the corresponding channel gain, and $\omega_{0}$ is the white noise power.

When a task is offloaded to the cloud server, at least one of the edge servers is selected as a relay node between the WD and the cloud server. We assume that the relay nodes for uplink and downlink transmissions can be different. Then, the one with the greatest uplink (downlink) transmission rate is selected as the uplink (downlink) relay node, as

(3) $$\begin{array}{cc} C_{nK+1}^{\mathrm{UL}}= & \max_{k\in K\setminus \{0,k+1\}}C_{nk}^{\mathrm{UL}},\\ C_{nK+1}^{\mathrm{DL}}= & \max_{k\in K\setminus \{0,k+1\}}C_{nk}^{\mathrm{DL}}. \end{array}$$

Moreover, there is neither uplink nor downlink transmission latency for local computing. For completeness, we also denote $C_{n0}^{\mathrm{UL}}=C_{n0}^{\mathrm{DL}}=\infty$.

Denote $T_{nm}^{\mathrm{UL}}$, $T_{nm}^{\mathrm{DL}}$ as the the uplink and downlink transmission latency for WD *n*’s *m*-th task, respectively. Then, we have

(4) $$\begin{array}{cc} T_{nm}^{\mathrm{UL}}= & \sum_{k\in K}\frac{\alpha_{nm}}{C_{nk}^{\mathrm{UL}}}a_{nmk},\\ T_{nm}^{\mathrm{DL}}= & \sum_{k\in K}\frac{\beta_{nm}}{C_{nk}^{\mathrm{DL}}}a_{nmk}, \end{array}$$

for n∈N and m∈M. Hence, the total communication delay for WD *n*’s *m*-th task can be expressed as

(5) $$\begin{array}{c} T_{nm}^{\mathrm{Comm}}=T_{nm}^{\mathrm{UL}}+T_{nm}^{\mathrm{DL}}+\tau a_{nmK+1}, \end{array}$$

where τ is constant representing the propagation delay between a edge server and the cloud server.

We also have the communication energy consumed by WD *n* for completing all *M* tasks as

(6) $$\begin{array}{c} E_{n}^{\mathrm{Comm}}=\sum_{m\in M}P_{n}^{\mathrm{TX}}T_{nm}^{\mathrm{UL}}+P_{n}^{\mathrm{RX}}T_{nm}^{\mathrm{DL}}, \end{array}$$

where $P_{n}^{\mathrm{RX}}$ is the corresponding reception power for WD *n*.

### 2.3. Computation Model

We denote $f_{k}$ as the number of CPU cycles for the server *k*. In general, the computation hardware at edge servers is more powerful than WDs, as $f_{0}\ll f_{k}\ll f_{K+1}$, for k∈K∖{0,K+1}. We assume that each server’s computational resources are equally shared among all tasks when two or more tasks are offloaded to the same server. For example, when two tasks are offloaded to the same server *k*, the computational resources allocated to each task are $f_{k}/2$. Then, the total number of CPU cycles allocated to WD *n*’s *m*-th task can be expressed as

(7) $$\begin{array}{c} f_{nm}=\sum_{k\in K}\frac{f_{k}a_{nmk}}{\sum_{n\in N}\sum_{m\in M}a_{nmk}}. \end{array}$$

Note that in real deployment of cloud computing systems, the allocated computational resources are smaller than $f_{nm}$ due to I/O interference between tasks at the same server [34].

Hence, the computation latency for WD *n*’s *m*-th task is

(8) $$\begin{array}{c} T_{nm}^{\mathrm{Comp}}=\frac{\gamma_{nm}}{f_{nm}}. \end{array}$$

Meanwhile, the energy consumed by WD *n* for completing all its *M* tasks can be expressed as

(9) $$\begin{array}{c} E_{n}^{Comp}=\kappa \gamma_{nm}f_{n0}^{2}\max_{m\in M}T_{nm}^{\mathrm{Comp}}a_{nm0}, \end{array}$$

where $\kappa =10^{-11}$ is the effective switched capacitance [35].

### 2.4. Problem Formulation

For both edge and cloud servers in MEC networks, energy is consumed whenever the server is turned on, which depends little on the number of tasks running on the servers. To reduce energy consumption at edge or cloud [36], some servers are preferred to be turned off when idle. Therefore, reducing communication energy or task processing energy at edge or cloud server is trivial. In this paper, we only consider energy consumption at WDs. To jointly evaluate the task completion latency and WDs’ energy consumption, we formulate the reward function as

(10) $$\begin{array}{c} Q\left(s,a\right)=\xi^{l}\max_{n\in N,m\in M}\left(T_{nm}^{\mathrm{Comm}}+T_{nm}^{\mathrm{Comp}}\right)+\xi^{e}\sum_{n\in N}\left(E_{n}^{\mathrm{Comm}}+E_{n}^{\mathrm{Comp}}\right), \end{array}$$

where $\xi^{l},\xi^{e}\in \left[0,1\right]$ are two scalar weights representing latency and energy consumption, respectively.

We consider a MEC network where WDs’ task requirements are time-varying, denoted as $s_{t}=\left\{{\left(d_{nm}^{UL},d_{nm}^{DL},d_{nm}^{WL}\right)}_{t}|n\in N,m\in M\right\}$. Given a system state $s_{t}$, we select an offloading action $a_{t}=\left\{{\left(a_{nmk}\right)}_{t}|n\in N,m\in M,k\in K\right\}$ from action space A following a policy $\pi (a_{t}|s_{t})$, and receive a scalar reward $r_{t}=Q\left(s_{t},a_{t}\right)$. This process continues with the increase of time index t=0,1,2,…,T. We aim to design a policy π which can efficiently generate an offloading action $a_{t}$ for each system state $s_{t}$ to minimize the expectation of the reward $r_{t}$, as

(11) $$\begin{array}{c} \lim_{T\to \infty }\frac{1}{T}\sum_{t=0}^{T}r_{t}. \end{array}$$

In general, this problem relates to the multi-armed bandit problem with NM arms and K+2 different options. Sometimes, it is referred as “trivial” [37] in the field of reinforcement learning since the reward function Q(s,a) is present. For example, given a system state s, we would always select the action with lowest value. However, searching for the optimal action within an action space with size ${\left(K+2\right)}^{NM}$ is time-consuming. In the next section, we study a linear programing relaxation-based (LR) approach to approximately generate the optimal action. Those important notations used throughout this paper are listed in Table 2.

## 3. Linear Programing Relaxation-Based Approach

In this section, we study a low-complexity algorithm to solve for the action with lowest reward value *Q*. Specifically, it takes the system state s as static variables and minimizes Q(s,a) with respect to the variables a, as

(12) $$\begin{array}{c} r=\min_{a\in A}Q(s,a). \end{array}$$

Since the algorithm does not use any previous state or action information, for brevity, we ignore the subscript *t* of all variables in this section. From (10), the action selection problem in (12) can be formulated as a general multi-objective optimization problem, which is expressed as follows:

(13a) $$\begin{array}{cc} \left(P1\right):\min_{a\in A}\;\;\;\; & \xi^{l}\max_{n\in N}\left(T_{nm}^{\mathrm{Comm}}+T_{nm}^{\mathrm{Comp}}\right)+\xi^{e}\sum_{n\in N}(\sum_{m\in M}\sum_{k\in K}\left(P_{n}^{\mathrm{TX}}\frac{\alpha_{nm}}{C_{nk}^{\mathrm{UL}}}+P_{n}^{\mathrm{RX}}\frac{\beta_{nm}}{C_{nk}^{\mathrm{DL}}}\right)a_{nmk}\\ & +\kappa \gamma_{nm}f_{n0}^{2}\max_{m\in M}T_{nm}^{\mathrm{Comp}}a_{nm0}) \end{array}$$

(13b) $$\begin{array}{cc} \mathrm{subject}\;\mathrm{to}:\; & a_{nmk}\in \left\{0,1\right\}, \end{array}$$

(13c) $$\begin{array}{c} \sum_{k\in K}a_{nmk}=1,\\ \forall n\in N,m\in M,k\in K. \end{array}$$

Problem (P1) is a three-dimensional integer programing problem whose solution space is in the size of $2^{NM(K+2)}$. Although solving for the optimal solution is computationally infeasible, lots of low-complexity heuristic algorithms can obtain near-optimal solutions. Here, we study a well-known LR-based algorithm [16,38] to solve (P1), which relaxes the binary variables $a_{nmk}\in \left\{0,1\right\}$ to real number $a_{nmk}\in \left[0,1\right]$. We introduce two new variables $y_{1},y_{2}\in R$ which are constrained by $y_{1}\geq \max_{n\in N}\left(T_{nm}^{\mathrm{Comm}}+T_{nm}^{\mathrm{Comp}}\right)$ and $y_{2}\geq \max_{m\in M}T_{nm}^{\mathrm{Comp}}$. From (5) and (8), problem (P1) can be transformed to be:

(14a) $$\begin{array}{cc} \left(P2\right):\min_{\begin{array}{c} a\in A\\ y_{1},y_{2}\in R \end{array}}\;\;\xi^{l}y_{1} & +\xi^{e}\sum_{n\in N}\left(\sum_{m\in M}\sum_{k\in K}\left(P_{n}^{\mathrm{TX}}\frac{\alpha_{nm}}{C_{nk}^{\mathrm{UL}}}+P_{n}^{\mathrm{RX}}\frac{\beta_{nm}}{C_{nk}^{\mathrm{DL}}}\right)a_{nmk}+\kappa \gamma_{nm}f_{n0}^{2}y_{2}a_{nm0}\right) \end{array}$$

(14b) $$\begin{array}{cc} \mathrm{subject}\;\mathrm{to}:\; & \sum_{k\in K}\left(\frac{\alpha_{nm}}{C_{nk}^{\mathrm{UL}}}a_{nmk}+\frac{\beta_{nm}}{C_{nk}^{\mathrm{DL}}}a_{nmk}\right)+\tau a_{nmK+1}+\frac{\gamma_{nm}}{f_{nm}}\leq y_{1}, \end{array}$$

(14c) $$\begin{array}{c} \frac{\gamma_{nm}}{f_{nm}}\leq y_{2}, \end{array}$$

(14d) $$\begin{array}{c} a_{nmk}\in \left[0,1\right], \end{array}$$

(14e) $$\begin{array}{c} \sum_{k\in K}a_{nmk}=1,\\ \forall n\in N,m\in M,k\in K. \end{array}$$

Here we propose a LR-based algorithm to solve for a feasible solution for problem (P1). We first solve problem (P2) via optimization tools for the optimal solution, denoted as $a^{*}$. Then, we recover binary characteristic of $a^{*}$ for a feasible solution for problem (P1). Considering the relaxed offloading decision sequence for WD *n*’s *m*-th task, $\left\{a_{nmk}^{*}|k\in K\right\}$, let $k_{nm}^{*}=\arg\max_{k\in K}a_{nmk}^{*}$ be the index of the maximum value $a_{nmk}^{*}$ among all K+2 decisions. Then, we choose $k_{nm}^{*}$ as the offloading server by setting $a_{nmk_{nm}^{*}}=1$ and $a_{nmk}=0$ for all those remaining $k\in K\setminus \{k_{nm}^{*}\}$. The procedure repeats till we obtain all binary offloading decision for all WDs’ tasks, a. We show the LR-based algorithm in Algorithm 1. Note that, in our simulation, (P2) is solved by a linear programming solver.

**Algorithm 1** Linear Programming Relaxation Approach-based Offloading Algorithm1:**Input:** $N,M,K,\alpha_{nm},\beta_{nm},\gamma_{nm},C_{nk}^{\mathrm{UL}},C_{nk}^{\mathrm{DL}},\forall n\in N,\forall m\in M,\forall k\in K$2:**Output:** a3:Solve (P2) to achieve $a^{*}$4:
**for**
n=1,2,…,N
**do**
5: **for**
m=1,2,…,M
**do**6:  $k_{nm}^{*}=\arg\max_{k\in K}a_{nmk}^{*}$;7:  $a_{nmk}=0,\forall k\in K\setminus \left\{k_{nm}^{*}\right\}$ and $a_{nmk_{nm}^{*}}=1$8: **end for**9:
**end for**


## 4. Deep Learning-Based Approach

In this section, we adopt a distributed deep learning-based offloading (DDLO) algorithm [23] to approximately minimize the expectation of reward presented in (11). By taking advantage of a batch of DNNs, the DDLO algorithm generates one binary offloading action from each DNN in a parallel way and chooses the action with the lowest reward as the output action.

**Figure 2:**
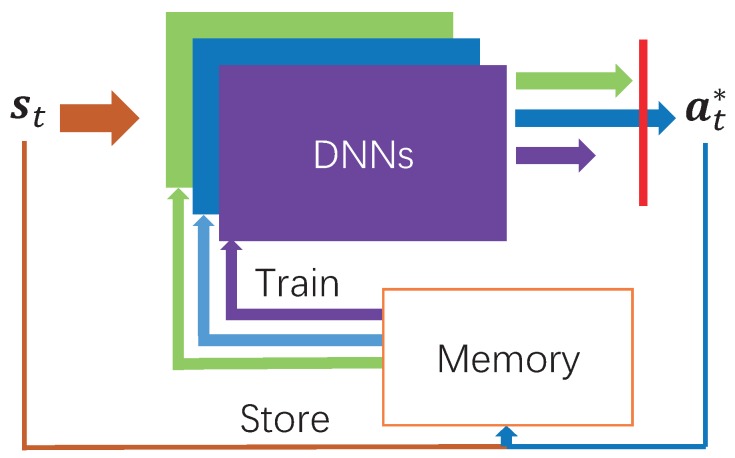
Architecture of distributed deep learning-based offloading (DDLO) [23].

The architecture of DDLO is illustrated in Figure 2, which is composed of *B* DNNs and a shared finite-sized memory structure. At each time slot *t*, it takes system state $s_{t}$ as the input and outputs a binary offloading decision $a_{t}^{*}$. Specifically, each DNN generates one candidate offloading action $a_{t}^{b}$, as

(15) $$\begin{array}{c} f_{\theta_{t}^{b}}:s_{t}\to a_{t}^{b}, \end{array}$$

where b∈B={1,2,…,B} is the index of the DNN and $f_{\theta_{t}^{b}}$ is a parameterized function representing the *b*-th DNN with parameters $\theta_{t}^{b}$. Among all those generated *B* candidates, the offloading action with the lowest reward is chosen as the output action, as

(16) $$\begin{array}{c} a_{t}^{*}=\underset{b\in B}{\arg\min}\;Q\left(s_{t},a_{t}^{b}\right). \end{array}$$

DDLO learns from its past experiences $\left(s_{t},a_{t}^{*}\right)$ to generate optimal offloading actions. At the beginning, all *B* DNNs are initialized with random parameter values $\theta_{0}^{b}$ and the memory is empty. Since different DNNs have different parameter values $\theta_{t}^{b}$, they will generate different offloading actions. By storing past experiences $\left(s_{t},a_{t}^{*}\right)$ in the memory, each DNN is trained and updated by randomly sampling a batch of training data from the memory. A gradient descent algorithm is performed to optimize parameter values $\theta_{t}^{b}$ of each DNN by minimizing the cross-entropy loss, as

$$\begin{array}{c} L\left(\theta_{t}^{b}\right)=-{a_{t}}^{T}\log f_{\theta_{t}^{b}}\left(s_{t}\right)-{\left(1-a_{t}\right)}^{T}\log\left(1-f_{\theta_{t}^{b}}\left(s_{t}\right)\right). \end{array}$$

In [23], all those *B* DNNs are assumed to be isomorphic. That is, they have the same number of layers and nodes and use the same activation function, Relu, at each hidden layer. In this paper, we further consider heterogeneous DDLO, where the hidden layers of all *B* DNNS are different. It is shown in Section 5.2 that heterogeneous DDLO can achieve better convergence performance than DDLO. We present our algorithm for multi-users, multi-tasks, multi-edges MEC networks in Algorithm 2.

**Algorithm 2** Heterogeneous DDLO for MEC networks 1:**Input:** all WDs’ task requirements $s_{t}$ 2:**Output:** offloading decision $a_{t}^{*}$ 3:
**Initialization:**
 4: Initialize all *B* DNNs with different random parameters $\theta_{t}^{b}$, b∈B; 5: Initialize memory structure with size *H*; 6:
**for**
t=1,2,…,G
**do**
 7: Input the same $s_{t}$ to each DNN. 8: Generate *B* offloading action candidates from the DNNs $\left\{a_{t}^{b}\right\}=f_{\theta_{t}^{b}}\left(s_{t}\right)$; 9: Select the offloading decision $a_{t}^{*}=\underset{b\in B}{\arg\min}\;Q\left(s_{t},a_{t}^{b}\right)$;  10: Store $\left(s_{t},a_{t}^{*}\right)$ into the memory structure;11: Randomly Sample *B* batches of training data from the memory structure;12: Train the DNNs;13:
**end for**


## 5. Performance Evaluation

### 5.1. Experiment Profile

In this section, we numerically study the performance of LR-based algorithm, DDLO (The source code of DDLO is available at https://github.com/revenol/DDLO.) algorithm, and heterogeneous DDLO algorithm for the MEC network. In the following simulations, we consider the CPU frequencies of each WD, each edge server, and the cloud server are $0.6\times 10^{9}$ cycles/s, $10\times 10^{9}$ cycles/s, and $1\times 10^{12}$ cycles/s, respectively [16]. Both the receiving power $P_{n}^{\mathrm{RX}}$ and the transmitting power $P_{n}^{\mathrm{TX}}$ of all WDs *n* are 0.2 W. When the *m*-th task of WD *n* is selected for offloading, the output data size after processing is assumed to be 20% of the input data size, $\beta_{nm}=0.2\alpha_{nm}$. We assume that the number of computational cycles required for each task is proportional to the input data size [35], as $\gamma_{nm}=q\alpha_{nm}$. Here the parameter *q* depends on different types of applications, whose values are listed in Table 3. For example, the Gzip application is labeled as type A with q=330 cycles/byte. In the following simulations, by default, we take type A application as an example to study different offloading algorithms. We assume that different WDs and edge servers are randomly distributed within a 30-by-30 ($m^{2}$) region following a Poisson point distribution with probability 3/10,100 and 1/400 for WDs and edges, respectively. The channel gain between WD *n* and edge *k* is calculated as $h_{nk}=103.8+20.9\times \log_{10}\left(d_{nk}\right)$[13], where $d_{nk}$ is the distance between WD *n* and edge *k*. The round-trip propagation delay between edge servers and cloud server is τ=15 ms. The bandwidth between WDs and edges is 10 M. The data size of each task is uniform distributed between 10 M and 20 M. The following simulation results are averaged over 100 realizations running on a server ThinkServer TD350 with Intel(R) Xeon(R) CPU E5-2620 v4 @ 2.1 Ghz processor.

To evaluate different offloading algorithms, we have pre-generated 30,000 input data according to the MEC network configurations. For each input data, we find the optimal offloading action by enumerating all $2^{NM(K+2)}$ combinations of binary offloading actions. For better illustrations, we study the reward ratio between the optimal offloading action and the ones generated from other algorithms, i.e., $\frac{optimal\;action}{action\;generated\;from\;algorithm}$. The closer the ratio is to 1, the better the generated offloading action.

### 5.2. Convergence Properties of Heterogeneous DDLO

**Figure 3:**
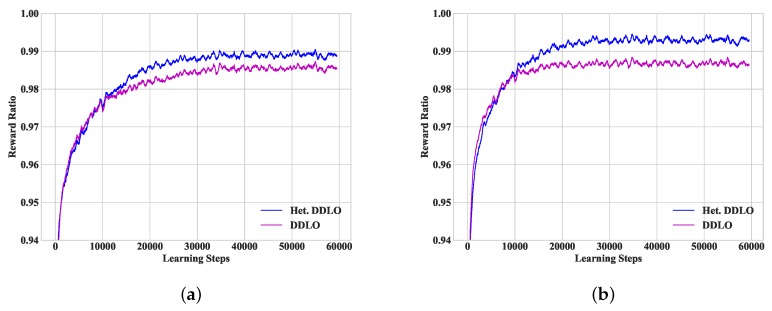
Convergence performance of DDLO and heterogeneous DDLO ((**a**) corresponds to the deep neural network (DNN) structure with two-hidden layers shown in Table 4; (**b**) corresponds to the DNN structure with three-hidden layers shown in Table 5).

To study the convergence performance of heterogeneous DDLO, we find the global optimal policy by enumerating all $2^{NM(K+2)}$ combinations of binary offloading policies and plot the ratio of the global optimal reward to the predicted results of heterogeneous DDLO. To restrict the enumerating space, we set the number of WDs N=3, the number of tasks for each user M=2, and the number of edge servers K=2. For both DDLO and heterogeneous DDLO evaluated in the following simulations, five fully connected DNNs are used in each algorithm. We study two-hidden-layer DNNs and three-hidden-layer DNNs for both DDLO and heterogeneous DDLO, whose structures are listed in Table 4 and Table 5, respectively. For fair comparison, we keep the interconnection complexity of each DNN in heterogeneous DDLO in the same scale of the one in DDLO. For example, in Table 4, the numbers of interconnections between two hidden layers of DNN1 are 120 × 80 = 9600 = 30 × 320 for both algorithms. In Figure 3, we compare the convergence performance of the heterogeneous DDLO algorithm with the DDLO algorithm [23]. In general, heterogeneous DDLO convergences faster and generates better offloading policy than DDLO. Intuitively, heterogeneous DDLO has higher degrees of exploration due to different DNN structures.

**Figure 4:**
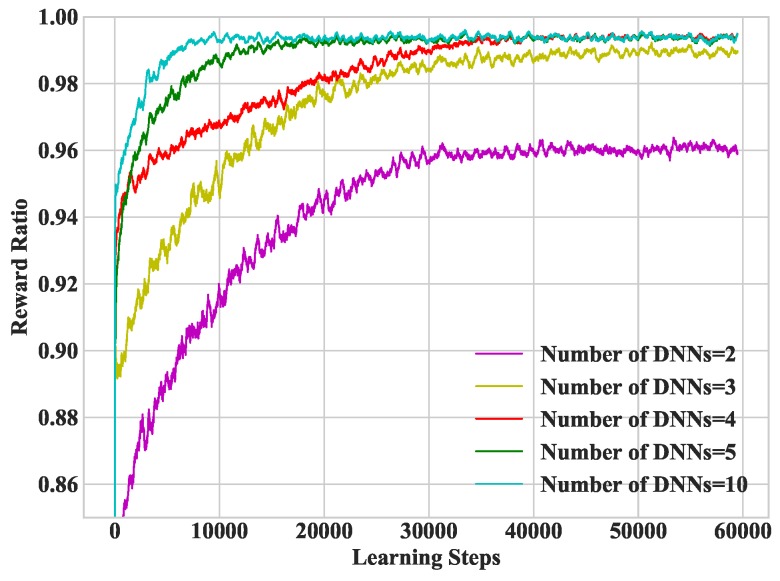
Convergence performance under different number of DNNs.

In Figure 4, we study heterogeneous DDLO under different number of DNNs. The more DNNs used, the faster heterogeneous DDLO converges, which requires more parallel computing resources. A small number of DNN may converge to local optimum, e.g., when the number of DNNS equals to 2. Note that, as reported in [23], DDLO cannot converge with a single DNN.

**Figure 5:**
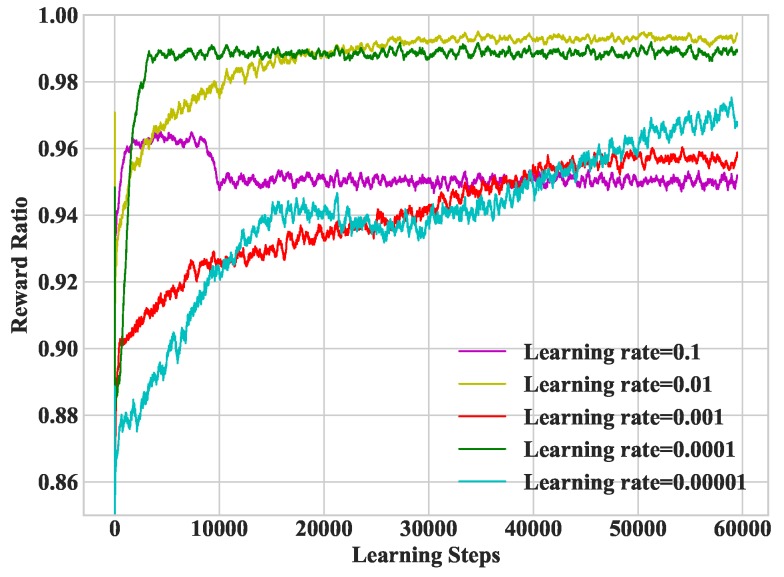
Convergence performance under different learning rates.

In Figure 5, we study heterogeneous DDLO under different learning rates. The larger the learning rate is, the faster the DNN convergence rate will be. However, it falls into the local optimal solution when the learning rate is too large, e.g., the learning rate is 0.1. Therefore, it is necessary to select an appropriate learning rate. In the following simulations, we set the learning rate as 0.01.

**Figure 6:**
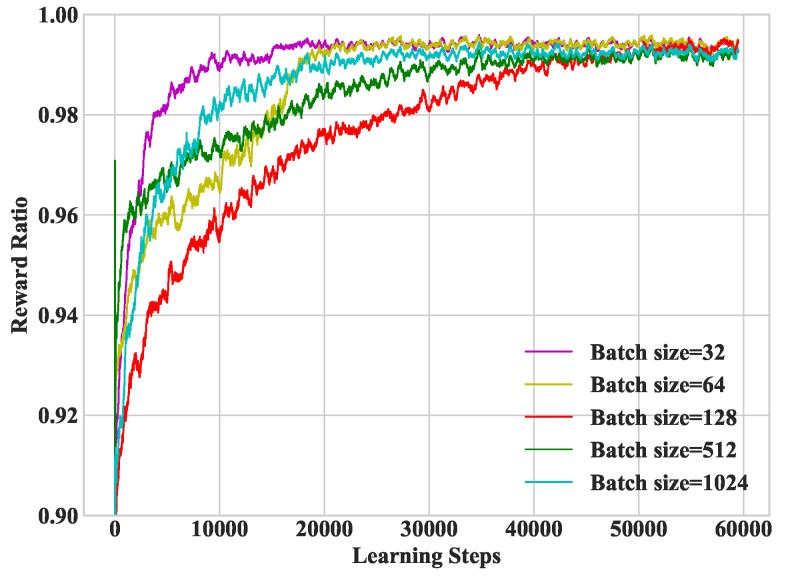
Convergence performance under different batch sizes.

In Figure 6, we study heterogeneous DDLO under different batch sizes. It refers to the number of training samples extracted from the memory in each training interval. From the numerical studies, we set the batch size as 32 in the following simulations.

**Figure 7:**
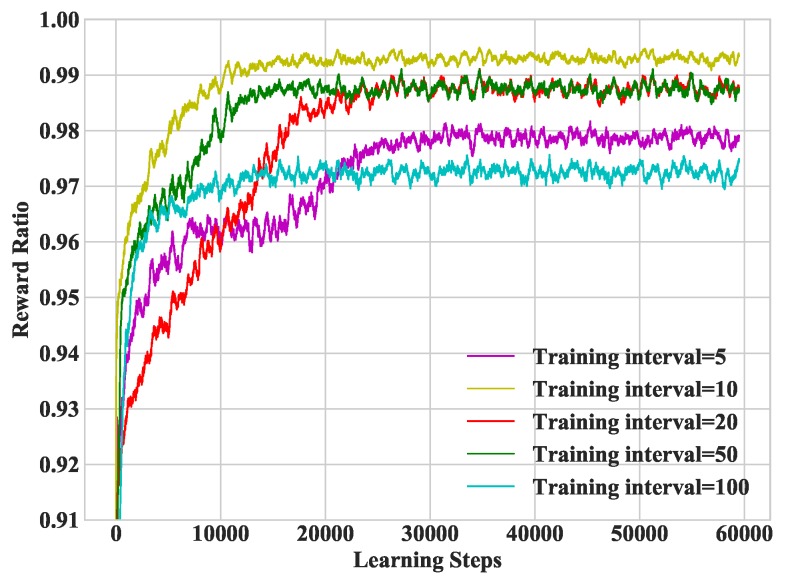
Convergence performance under different training intervals.

In Figure 7, we study heterogeneous DDLO under different training intervals. As a matter of fact, the training interval cannot be too small. In the following simulations, we set the training interval as 10.

### 5.3. Performance of Different Offloading Policies

**Figure 8:**
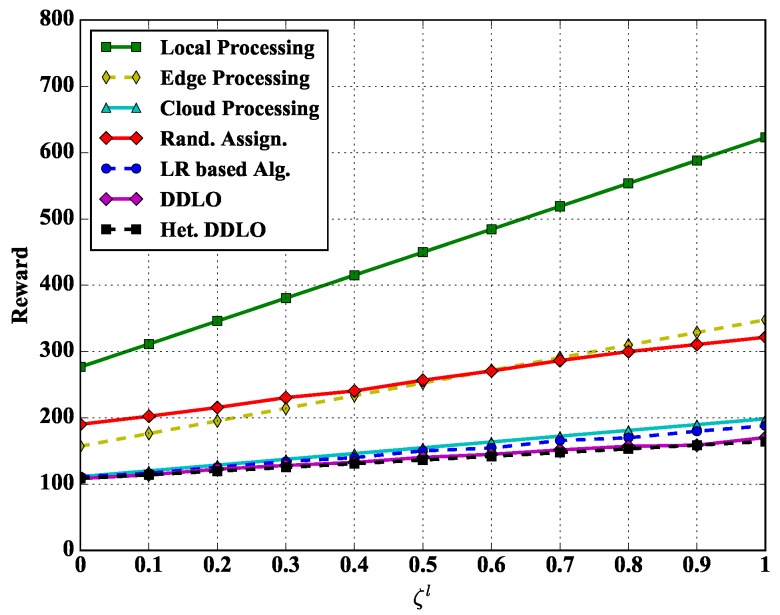
Algorithm comparison under different $\xi^{l}$.

**Figure 9:**
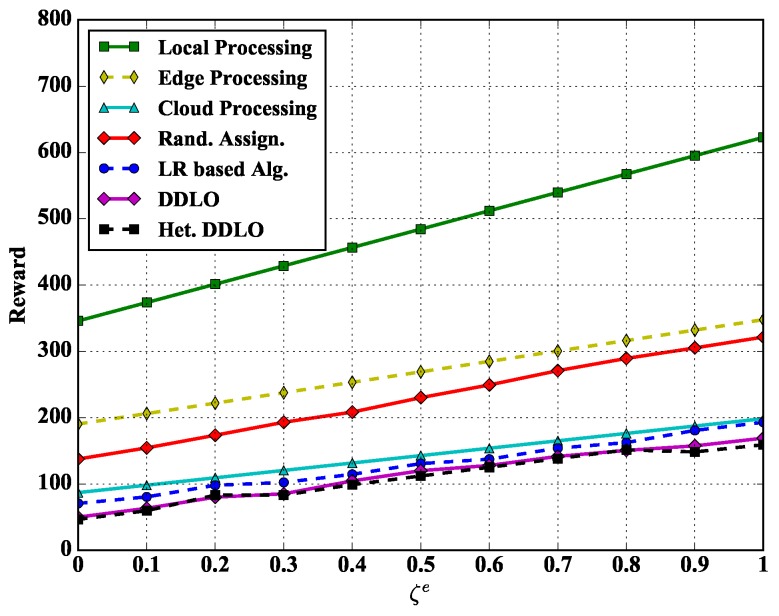
Algorithm comparison under different $\xi^{e}$.

We study the reward performance of different policies under different weights $\xi^{l}$ and $\xi^{e}$ in Figure 8 and Figure 9. Regarding to the weighted sum energy consumption and latency performance, we also evaluate other four representative benchmarks:

- *Edge Processing.* All tasks are offloaded to and processed at edge servers, i.e., setting $\sum_{k=1}^{K}a_{nmk}=1$, n∈N, m∈M.
- *Cloud Processing.* All tasks are offloaded to and processed at could server, i.e., setting $a_{nmK+1}=1$, n∈N, m∈M.
- *Local Processing.* All tasks are processed locally at WDs, i.e., setting $a_{nm0}=1$, n∈N, m∈M.
- *Random Assignment.* Offloading decisions are generated randomly.

We set the energy scalar and latency scalars as constants $\xi^{e}=1$ and $\xi^{l}=1$ in Figure 8 and Figure 9, respectively. With the increase of delay scalar $\xi^{l}$ and $\xi^{e}$, the reward values of all policies increase. The Local Processing policy generates largest reward while both DDLO and heterogeneous DDLO outperform other offloading policies. When $\xi^{e}=0$, the system reward only considers the latency, and the Cloud Processing policy takes longer time than other integer offloading policies, e.g., LR-based algorithm and heterogeneous DDLO.

### 5.4. Impacts of Different MEC Network Structures

**Figure 10:**
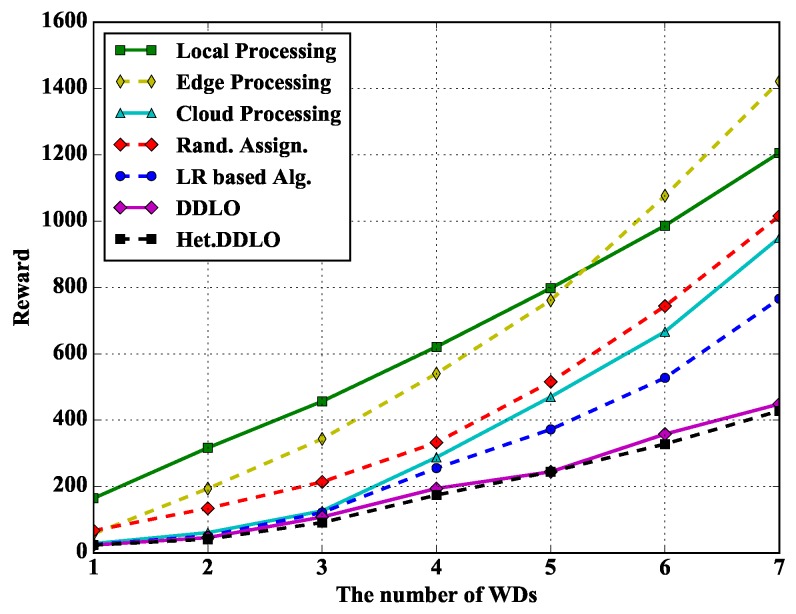
Algorithm comparison under different number of WDs when $\xi^{l}=1$ and $\xi^{e}=0.4$.

In Figure 10, we study the performance of different policies under different number of WDs. Heterogeneous DDLO outperforms LR-based algorithm. With the increasing number of WDs, the total reward of Edge Processing policy grows faster than other offloading policies because more users will jointly occupy one edge’s resources, resulting a low processing speed.

**Figure 11:**
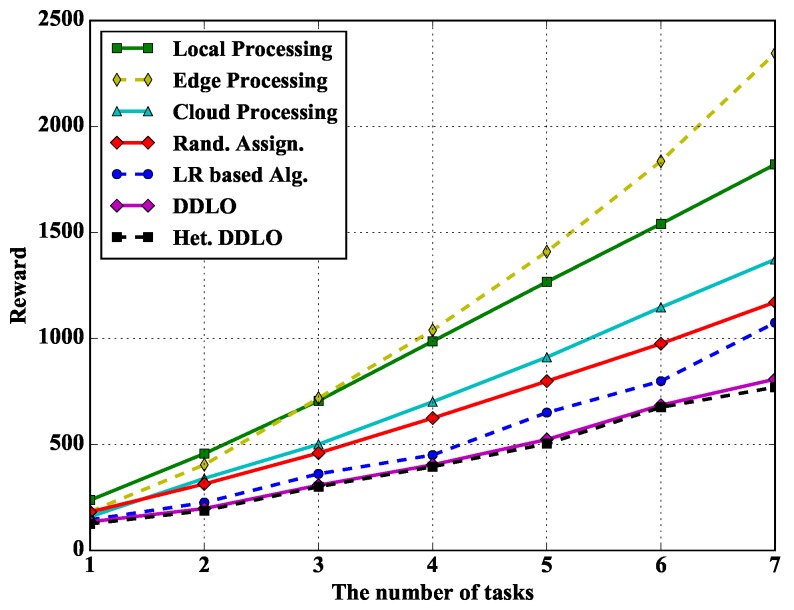
Algorithm comparison under different number of tasks when $\xi^{l}=1$ and $\xi^{e}=0.4$.

In Figure 11, we study the performance of different policies under different number of tasks. With the increase of the number of tasks, the total reward of Edge Processing policy grows faster and faster. Because when an edge server processes multiple tasks at the same time, its processing units are shared among all tasks. DDLO and heterogeneous DDLO outperform other offloading policies.

**Figure 12:**
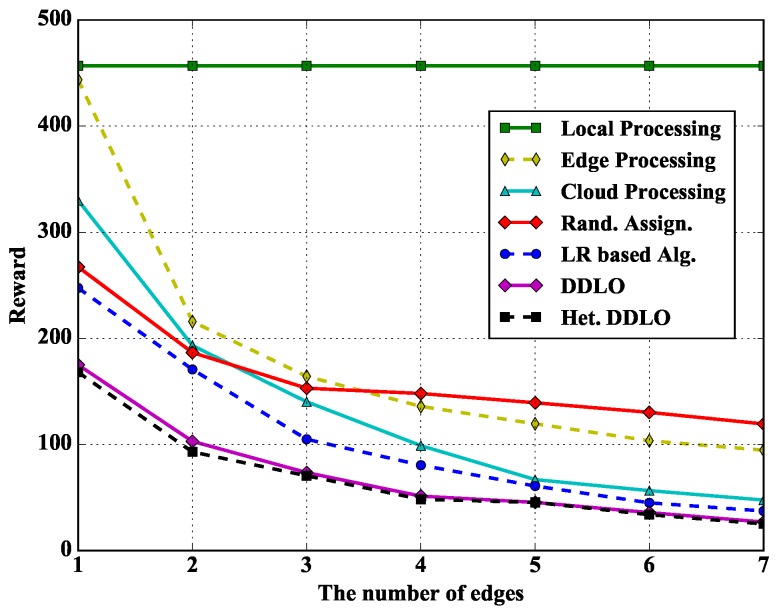
Algorithm comparison under different number of edges when $\xi^{l}=1$ and $\xi^{e}=0.4$.

In Figure 12, we study the performance of different policies under different number of edges. The Local Processing policy does not change with the number of edges. The reward of other policies gradually decreases with the increase of edge servers due to more processing resources and likely closer proximity to WDs.

### 5.5. Impacts of Different Types of Applications

**Figure 13:**
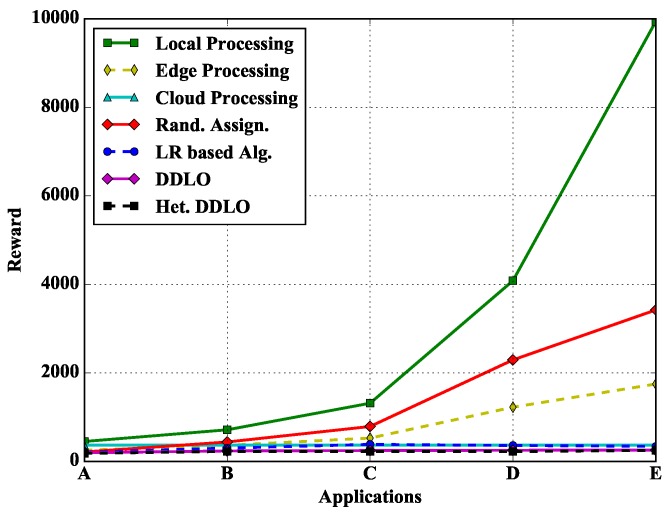
Algorithm comparison under different types of applications when $\xi^{l}=1$ and $\xi^{e}=0.4$.

In Figure 13, we study the performance of different policies under different types of applications. Because there are plenty of computing resources at the cloud server, the total cost of all cloud computing will not change when the application type is changed. Both local and edge computing need to consider the computing delay, and the computing delay is directly positively correlated with *q*, while the energy consumption is correlated with time delay. Therefore, when the application type changes and *q* increases, the total cost of local and edge computing will also increase. The optimization algorithm will choose cloud processing more, so its total cost grows very slowly.

### 5.6. Computation Time

In Table 6, we compare the CPU computation time between heterogeneous DDLO algorithm and LR-based algorithm under different number of WDs. Heterogeneous DDLO generates one offloading decision within one millisecond (Note that the CPU computation time of heterogeneous DDLO in this paper is much less than the one of DDLO presented in [23] since resource allocation is not considered here.), which is several orders faster than LR-based algorithm. Furthermore, the computation time of heterogeneous DDLO algorithm is insensitive to the number of WDs. For example, it increases from 0.63 millisecond to 0.74 millisecond when the number of WDs increases from 1 to 7. In comparison, the LR-based algorithm increases by 1641%, from 0.33 second to 5.8 seconds, which is inapplicable for real-time applications.

## 6. Conclusions

In this work, we studied multi-server multi-user multi-task computation offloading for MEC networks, with the aim to guarantee the network’s quality of service and to minimize WDs’ energy consumption. By formulating different real-time task offloading decisions as static optimization problems, we investigated a LR-based algorithm to approximate the optimum. By taking advantage of deep reinforcement learning, we further investigated the heterogeneous DDLO algorithm for MEC networks. Numerical results show that both algorithms can achieve better performance than other offloading decisions, e.g., Local Processing algorithm, Edge Processing algorithm, and Cloud Processing algorithm. Furthermore, the heterogeneous DDLO outperforms the LR-based algorithm by generating better performance and consuming several orders less computation time. Specifically, the heterogeneous DDLO generates one offloading decision in less than 1 millisecond, which is insensitive to the number of WDs.

## Figures and Tables

**Table 1:** Related works on computation offloading in mobile edge computing (MEC) networks.

Publication	Task		User		Edge Server		Remote Server
	Single	Multiple	Single	Multiple	Single	Multiple	
Liu et al. [12]	*√*			*√*	*√*		*√*
Bi et al. [14]	*√*			*√*	*√*		
Dinh et al. [16]		*√*	*√*			*√*	
Huang et al. [23]		*√*		*√*	*√*		
Wei et al. [24]		*√*	*√*		*√*		
You et al. [25]	*√*		*√*		*√*		
Munoz et al. [26]	*√*		*√*		*√*		
Huang et al. [27]	*√*			*√*	*√*		
Chen et al. [28]	*√*			*√*	*√*		
Wang et al. [29]	*√*		*√*			*√*	
Dinh et al. [30]	*√*			*√*		*√*	
You et al. [33]	*√*			*√*	*√*		
Chen et al. [31]		*√*		*√*	*√*		*√*
Li et al. [32]	*√*			*√*		*√*	*√*
Our Work		*√*		*√*		*√*	*√*

**Table 2:** Notations used in this paper.

Notation	Definition
$a_{nmk}$	$a_{nmk}=1$ if WD *n* offloads its task *m* to the server *k*. Otherwise, $a_{nmk}=0$
$\alpha_{nm}$	Input date size of the task *m* of WD *n*
$\beta_{nm}$	Output date size of the task *m* of WD *n*
$\gamma_{nm}$	The number of CPU cycles to process the task *m* of WD *n*
$C_{nk}^{\mathrm{UL}}$, $C_{nk}^{\mathrm{DL}}$	Transmission rates between WD *n* and edge server *k*
$B_{nk}^{\mathrm{UL}}$, $B_{nk}^{\mathrm{DL}}$	Transmission channel bandwidths between WD *n* and edge server *k*
$P_{n}^{TX}$, $P_{k}^{TX}$	Transmission powers of WD *n* and edge server *k*
$h_{nk}^{\mathrm{UL}}$,$h_{nk}^{\mathrm{DL}}$	Transmission channel gains between WD *n* and edge server *k*
$\omega_{0}$	The white noise power level
$T_{nmk}^{\mathrm{UL}}$, $T_{nmk}^{\mathrm{DL}}$	Uplink and downlink transmission latency of the task *m* of WD *n* to server *k*
τ	Transmission latency between a edge server and the cloud server
$T_{n}^{\mathrm{Comm}}$	Total transmission latency of WD *n*
$\rho^{t}$	Transmission power
$E_{n}^{\mathrm{Comm}}$	Total transmission energy consumption of WD *n*
$f_{k}$	Clock frequency of CPU *k*
$T_{nmk}^{\mathrm{Comp}}$, $T_{nm0}^{\mathrm{Comp}}$	Computing latency of the task *m* of WD *n* in edge server *k* or locally
$T_{n}^{\mathrm{Comp}}$	Total computing latency of WD *n*
κ	Effective switched capacitance
$E_{nm}^{\mathrm{Comp}}$	Computing energy consumption of WD *n*’s *m*-th task
$E_{n}^{\mathrm{Comp}}$	Total computing energy consumption of WD *n*
$\xi^{l}$	Scalar weights of latency
$\xi^{e}$	Scalar weights of energy consumption

**Table 3:** Application complexity [30,35].

Application	Labels	Computation to Data Ratio *q* (Cycles Per Byte)
Gzip	A	330
pdf2text(N900 data sheet)	B	960
z264 CBR encode	C	1900
html2text	D	5900
pdf2text(E72 data sheet)	E	8900

**Table 4:** DNN structures used in DDLO and heterogeneous DDLO with 2 hidden layers.

DNNs	Number of Neurons in DDLO				Number of Neurons in Het. DDLO			
	Input	1st Hidden	2nd Hidden	Output	Input	1st Hidden	2nd Hidden	Output
DNN 1	6	120	80	24	6	30	320	24
DNN 2	6	120	80	24	6	60	160	24
DNN 3	6	120	80	24	6	120	80	24
DNN 4	6	120	80	24	6	240	40	24
DNN 5	6	120	80	24	6	480	20	24

**Table 5:** DNN structures used in DDLO and heterogeneous DDLO with 3 hidden layers.

DNNs	Number of Neurons in DDLO					Number of Neurons in Het. DDLO				
	Input	1st Hidden	2nd Hidden	3th Hidden	Output	Input	1st Hidden	2nd Hidden	3th Hidden	Output
DNN 1	6	80	60	40	24	6	320	60	10	24
DNN 2	6	80	60	40	24	6	160	60	20	24
DNN 3	6	80	60	40	24	6	80	60	40	24
DNN 4	6	80	60	40	24	6	40	60	80	24
DNN 5	6	80	60	40	24	6	20	60	160	24

**Table 6:** Average CPU computation time under various number of WDs.

Number of WDs	DDLO (s)	Het. DDLO (s)	LR-based Alg. (s)
1	6.11 × $10^{-4}$	6.28 × $10^{-4}$	3.30 × $10^{-1}$
2	6.42 × $10^{-4}$	6.47 × $10^{-4}$	9.66 × $10^{-1}$
3	6.69 × $10^{-4}$	6.67 × $10^{-4}$	1.68
4	6.88 × $10^{-4}$	6.82 × $10^{-4}$	2.41
5	6.99 × $10^{-4}$	7.02 × $10^{-4}$	3.66
6	7.19 × $10^{-4}$	7.20 × $10^{-4}$	4.41
7	7.36 × $10^{-4}$	7.39 × $10^{-4}$	5.75
